# Upconverted Hot Electrons and Solvated Electrons from Mn-Doped Semiconductor Nanocrystals for Photochemistry: Perspective

**DOI:** 10.1021/acs.jpclett.5c03858

**Published:** 2026-01-20

**Authors:** Connor Orrison, Ian Murray, Dong Hee Son

**Affiliations:** † Department of Chemistry, 14736 Texas A&M University, College Station, Texas 77843, United States; ‡ Department of Physics and Astronomy, Texas A&M University, College Station, Texas 77843, United States; § Center for Nanomedicine, Institute for Basic Science and Graduate Program of Nano Biomedical Engineering, Advanced Science Institute, Yonsei University, Seoul 03722, Republic of Korea

## Abstract

Hot electrons photogenerated in semiconductor nanocrystals enable powerful redox reactivity due to their large excess energy and their capability for long-range transfer over high energy barriers. Among various strategies for hot electron generation, Mn-mediated Auger upconversion in Mn-doped semiconductor nanocrystals has emerged as a particularly effective method. This process generates hot electrons with large excess energies, a fraction of which can even exceed the vacuum level, enabling their emission as free electrons into the vacuum or their injection into surrounding liquid media to form reactive solvated electrons. These unique properties of the upconverted hot electrons open new pathways for driving thermodynamically and kinetically demanding reactions in various liquid media. This Perspective discusses recent advances in Mn-mediated hot electron upconversion, diverse chemical transformations enabled by upconverted hot electrons, and the important issues that need to be addressed to harness the full potential of upconverted hot electron-driven photochemistry.

## Graphical abstract

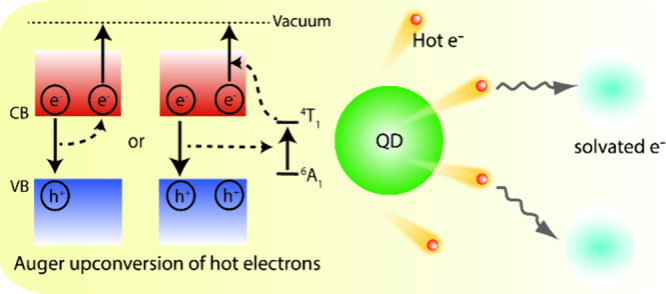

Photogenerated hot electrons carrying excess kinetic energy above the conduction bandedge in semiconductor nanocrystals have been of much interest due to their benefits in photovoltaics^1–5^ and photocatalysis.^6–10^ Since their excess energy provides a higher reduction potential and enables more facile electron transfer across energy barriers over long distances, hot electrons have significant advantages over the thermalized electrons for such applications.^11^ Furthermore, sufficiently energetic hot electrons can produce solvated electrons when injected into liquid solvent media, enabling solvated electron-induced reduction reactions.^12–18^ Among various ways of producing hot electrons in semiconductor nanocrystals, Mn-mediated Auger-upconversion in Mn-doped semiconductor nanocrystals has received significant attention due to its capability to produce energetic hot electrons under weak visible light with high efficiency. The long-lived Mn intermediate state (e.g., many ms) enables efficient hot electron generation even under weak continuous wave (cw) excitation. The large excess energy (>2 eV) of the upconverted hot electrons is sufficient to produce solvated electrons in various solvents, in addition to enabling interfacial hot electron transfer. These benefits of Mn-mediated Auger-upconverted hot electrons have been demonstrated in various photochemical reactions.^19–23^ This Perspective provides an overview of recent progress in the generation of hot electrons and solvated electrons via Mn-mediated Auger upconversion and their applications in photochemical reactions as well as the important issues the future studies will need to address for utilization of this process for visible-light-driven hot electron and solvated electron photochemistry.

Hot electron generation via Auger processes in semiconductor nanocrystals can occur via several different pathways depending on the electronic states involved in the Auger process. Here, we compare different Auger processes explored in both undoped and Mn-doped semiconductor nanocrystals, as shown in Figure 1 and emphasize the advantage of involving doped Mn^2+^ in the Auger process to produce hot electrons. In a conventional multiexciton Auger process in undoped semiconductor nanocrystals, the energy from the recombination of one exciton is transferred to the second exciton resulting in the creation of a hot electron (Figure 1a). This process is responsible for the well-known charging of the nanocrystals via Auger ionization.^24^ However, because biexciton Auger recombination requires multiexciton generation within the lifetime of an exciton, it often requires high-intensity pulsed excitation limiting its practicality for the applications of hot electrons.^25,26^

**Figure 1:**
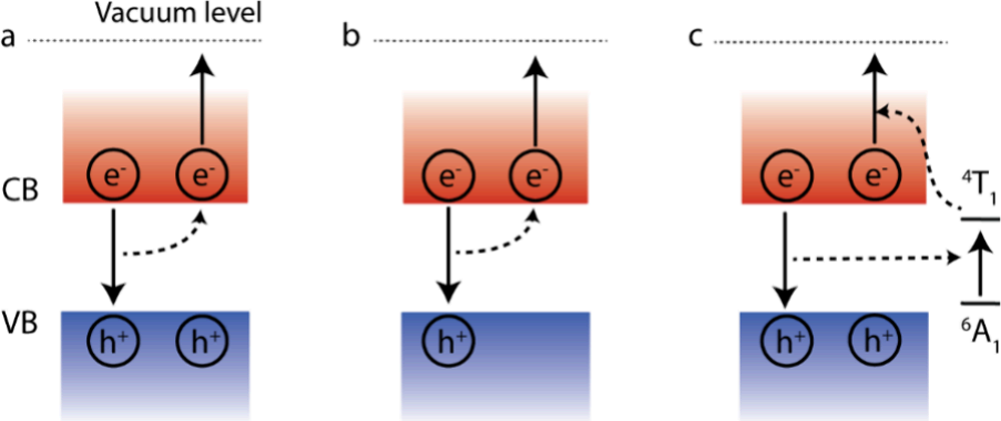
Methods of photogeneration of hot electrons in semiconductor nanocrystals. CB: conduction band; VB: valence band. (a) Auger recombination of biexciton, (b) Negative trion recombination, and (c) Auger upconversion via Mn excited state (^4^T_1_) as an intermediate state.

To circumvent the disadvantage of the multiexciton Auger process in undoped semiconductor nanocrystals as the mechanism to produce hot electrons, Auger processes involving longer-lived electronic states have been explored. One approach is realizing the Auger process between one exciton and one extra electron in the conduction band, as illustrated in Figure 1b. The extra electron in the negatively charged semiconductor nanocrystals lacks a hole for the electron to recombine with, therefore it survives significantly longer than a typical exciton. The Auger process between the extra electron and an additionally photoexcited exciton produces a hot electron, which can be viewed as the Auger recombination of a negative trion, which has been reported to occur on tens to ns time scale depending on the size of the nanocrystals.^27–29^ Because of the longer lifetime of an extra electron, the Auger recombination of a negative trion is more effective for hot electron generation than biexciton Auger recombination, although trion recombination is generally slower than biexciton recombination. Negatively charged semiconductor nanocrystals have been prepared in several different ways. One is removing the hole after photoexcitation of one exciton with a hole scavenger.^28–31^ This has been achieved using molecular hole scavengers or redox-active ligands.^28–34^ Hole removal has also been achieved by doping other elements within the nanocrystals, which can localize the photogenerated holes. Dopants such as Cu can also serve as internal hole traps.^35–37^ Direct injection of an electron into the conduction band via chemical charging or electrochemical charging is another strategy that has been used to create negative trion.^38–40^

In Mn-mediated Auger upconversion of hot electrons, the combination of a long-lived ligand field state of Mn^2+^ combined with rapid energy exchange between the exciton and Mn^2+^ is what renders it a more efficient hot electron-generating mechanism compared to other Auger processes. The initial excited exciton in Mn-doped nanocrystals undergoes rapid energy transfer to Mn^2+^ on ultrafast time scale (subps to a few ps) populating the lowest excited ligand field state of Mn (^4^T_1_) as illustrated in Figure 1c. The lifetime of ^4^T_1_ state of Mn^2+^ can be several ms due to the forbidden nature of the transition, while it varies depending on the host and doping level.^41–44^ Therefore, the excited Mn^2+^ state serves as a long-lived energy reservoir that stores >2 eV of energy. Upon photoexcitation of another exciton within the lifetime of the ^4^T_1_ state, Auger energy transfer from the ^4^T_1_ state of Mn^2+^ to the exciton produces a hot electron. Because of the long lifetime of the ^4^T_1_ state, cw excitation at intensities comparable to concentrated solar radiation is sufficient to enable the Mn-mediated Auger upconversion of hot electrons. Since the Auger process involves energy exchange between the exciton (or electron) and the forbidden transition localized on Mn^2+^, the rate of energy exchange is also a crucial factor in determining the efficiency of hot electron generation. In the case of II–VI host nanocrystals doped with Mn^2+^, the initial energy transfer from exciton to Mn^2+^ was reported to occur on a time scale of sub ps to tens of ps depending on the structural details of the doped nanocrystals.^43^ Both the Mn^2+^ excited state lifetime and the energy transfer rate depend strongly on host composition, dopant position, and local coordination. Currently, the qualitative relationship between the energy transfer rate and doping density and location as well as the degree of quantum confinement of the host nanocrystals is reasonably well understood for a given system. However, developing accurate, quantitative predictions from theory remains challenging due to the complexity of dopant-host electronic coupling in realistic nanocrystals, which often have heterogeneous dopant placement and defects that can localize the exciton (or carrier) wave function. Figure 2 shows the clear trends in the dependence of the energy transfer rate on the radial doping location and doping density in Mn-doped CdS/ZnS core/shell quantum dots (QDs), where these two parameters were independently varied as orthogonal variables.^45^ The energy transfer rate increases with the radial doping location closer to the center of the nanocrystals and with increasing doping density at a given radial doping location. This was rationalized in terms of exciton-dopant wave function overlap that determines the magnitude of electronic interaction mediating the energy transfer. However, the strong dependence of the energy transfer rate on the chemical identity of the host nanocrystals discussed in the next section is still not fully understood and requires further study.

**Figure 2:**
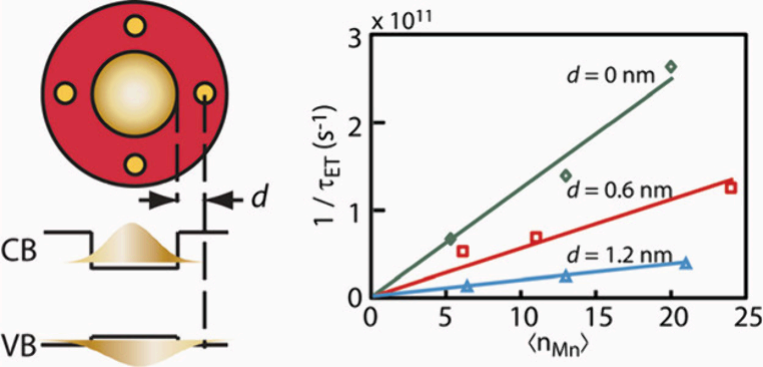
Doping location dependent energy transfer rate constant (1/τ_ET_). From ref 43 (Copyright 2012, American Chemical Society).

The earlier demonstration of Mn-mediated hot electron upconversion was made in II–VI host semiconductor nanocrystals with bandgap exceeding the accepting ligand field transition energy of Mn^2+^ under cw excitation.^19,38,44,46–48^ If the bandgap of the host falls below the ligand field transition energy of Mn^2+^, the characteristic Mn emission centered at ∼600 nm indicating exciton-Mn energy transfer was not observed, which prevents hot electron upconversion under cw excitation conditions.^49^ Recently, the Auger upconversion of hot electrons in Mn-doped CdSe nanocrystals with host bandgap in near resonance with the ligand field transition of Mn^2+^ was reported under pulsed excitation condition that excites multiple excitons simultaneously in each nanocrystal, although cw excitation would not be efficient.^50,51^ Subps energy transfer (200–300 fs) between exciton and dopant in this system, which was described as spin-conserving spin-exchange energy transfer, enabled the Mn-mediated hot electron Auger upconversion despite the very small steady-state Mn^2+^ excited state population under cw excitation. Participation of two excited Mn^2+^ centers in the Auger process was also proposed under near-bandedge excitation.^50^

While most demonstrations of Mn-mediated hot electron upconversion have been in Mn-doped II–VI semiconductor nanocrystals, other host materials have also been investigated. Recently, Mn-doped lead halide perovskite (APbX_3_) nanocrystals have been explored as host materials for Mn-mediated hot-electron upconversion.^52–58^ The first Mn-doped metal halide perovskite nanocrystals that showed the Auger upconversion of hot electrons are Mn-doped CsPbCl_3_ and CsPbBr_3_ nanocrystals.^59^ Stronger quantum confinement in CsPbBr_3_ nanoplatelets enhances the exciton-Mn energy transfer and hot electron generation relative to nanocubes. These results suggest that Mn-mediated Auger-upconversion can be achieved in a wider range of host nanocrystals universally provided that the host nanocrystals support the long-lived sensitized dopant excited state and rapid energy exchange between the host and dopant.

The efficiency of generating Auger-upconverted hot electrons in Mn-doped QDs depends on the efficiency of each step of the upconversion process, which competes with other dynamic processes, i.e., radiative and nonradiative decay of exciton and nonradiative decay of the Mn^2+^ excited state. Experimentally, the first step (exciton-Mn sensitization) is readily measured by ultrafast spectroscopy, whereas the second step (Auger back-transfer from excited Mn^2+^) remains difficult to directly quantify. To date, the rates of exciton-Mn energy transfer in several different groups of host semiconductor nanocrystals have been investigated including II–VI (CdS/ZnS, CdSe),^43,45,50,51,60–63^ metal halide perovskite (CsPbX_3_)^53,64–72^ and InP nanocrystals.^73,74^ The energy transfer exhibits significant dependence on the host material, with II–VI nanocrystals generally being the most effective followed by lead halide perovskite and InP nanocrystals, as summarized in Table 1. Reported energy transfer times range from ∼100 fs in II–VI hosts to hundreds of ns in InP/ZnS nanocrystals. While these results clearly indicate a strong dependence on the host material, the detailed role of host composition in governing the rate of energy transfer from photogenerated excitons to Mn^2+^ dopants is not yet fully understood.

**Table 1:** Energy Transfer Times in Various Mn-Doped Semiconductor Quantum Dots (QD) and Nanoplatelets (NPL)

Host material, size, ligands	Doping level and location	Energy transfer time	ref.
CdSe/CdS QD, 2.3–4.6 nm core, ∼1 nm shell, oleate	∼0.5–1 Mn/QD	140 fs (core doping)	^51^
		2.6 ps (core surface doping)	
CdSe/CdS QD, 3.75 nm core, 4.7 nm with shell, oleate	2.1 mol % (13 Mn/QD)	∼100 fs	^50^
CdSe NPL, ∼1.4 × 19 × 6 nm^3^, oleate	16–60 Mn/QD	2.6 ps (16 Mn/QD)	^60^
		1.3 ps (51 Mn/QD)	
CdS/ZnS QD, 3.2 nm core, ∼5 nm including shell, oleylamine	6, 12, and 22 Mn/QD, doped in the shell radially with d = 0–1.2 nm from the core/shell interface	d = 0 nm, 22–3.8 ps	^43^
		d = 0.6 nm, 62.5–16.7 ps	
		d = 1.2 nm, 200–66.7 ps	
CdS/ZnS QD, 3 nm core, 5 nm including shell, oleylamine	0, 2, 3, 4, and 13 Mn/QD, doped 0.6 nm from core in ZnS shell	∼60 ps	^45^
ZnSe QDs, 4.3 nm, oleate	15 mol %	∼100 fs	^61^
ZnSe/(Zn,Mn)Se quantum wells, (Zn,Mn)Se thin films	23 mol %	∼15 ps	^62^
ZnS QDs, 6 nm	2 mol %	∼700 ps	^63^
CsPbCl_3_ QDs, ∼7–10 nm, oleylamine and oleate	0.2 mol %	3.6 ns (from PL data)	^53^
CsPbCl_3_ QDs, 10 nm, oleylamine and oleate	0.4 mol %	380 ps	^64^
CsPbCl_3_ QDs, 8 nm, oleylamine and oleate	∼4 mol %	ns-ps	^65^
CsPbCl_3_ QDs, ∼6.6 nm	∼3.9 mol %	1.65 ns	^67^
CsPbCl_3_ QDs, ∼8 nm, oleylamine and oleate	∼4 mol %	∼303 ps	^66^
CsPbCl_3_ QDs, 11 nm, oleylamine and oleate	9.6 mol %	∼3–8 ns	^68^
CsPbCl_0.7_Br_0.3_, oleylamine and oleate	13 mol %	22.7 ps	^71^
CsPbCl_3‑x_Br_*x*_, 12.5 nm, oleylamine and oleate	∼1 mol %	1–10 ns	^69^
CsPbBr_3_ NPL, 3 × 68 × 30 nm, oleylamine and oleate	N/A	<1 ps	^72^
InP/ZnS QD, 0.8 nm core, 3.4 nm with shell, oleylamine	∼9 mol %	∼51 ps	^74^
InP/ZnS QD, 2 nm core, 4 nm shell, oleylamine	14 and 38%, shell doping	588 and 204 ns	^73^

Reported energy transfer rates are obtained from nanocrystals with varying sizes, dopant densities, and spatial distributions, all of which directly affect the energy transfer rate and complicate direct cross-system comparisons. Moreover, pump–probe measurements are inherently excitation fluence-dependent; therefore, the reported rates require careful interpretation. While exciton-Mn energy transfer is not the sole factor determining hot electron generation efficiency, it is a useful guiding metric for designing hot electron-generating nanocrystals because the electronic interactions mediating both steps of Mn-driven upconversion are closely related.

Several different methods have been employed for the detection and characterization of upconverted hot electrons from Mn-doped semiconductor nanocrystals. The most direct way of detecting hot electrons is to measure photoelectron emission in vacuum that detects the subpopulation of hot electrons lying above the vacuum level.^47,59,75,76^ Another method is to measure the photocurrent across a tunneling barrier that blocks the bandedge electrons and selectively detecting hot electrons.^38,48^ Hot electrons have also been indirectly detected through either the detection of solvated electrons formed from hot electrons ejected into the solvent medium or through the use of reduction reaction that has sufficiently high reduction potential to selectively report the hot electrons.^21,50,61^

Figure 3a shows how the hot electrons ejected above vacuum level from Mn-doped nanocrystals are detected as the photoemission current using a setup resembling a diode vacuum tube, first reported by Dong et al.^47^ Because the higher-energy subpopulation of the hot electrons above the vacuum level constitutes only a small fraction of the hot electron population, the current measured cannot be directly compared between different samples to assess the efficiency of hot electron upconversion. However, this approach provides the most direct detection of hot electrons and thus offers useful insights into hot electron properties, as described below. Figure 3b shows the excitation intensity dependence of hot electron photoemission current under cw excitation conditions measured from Mn-doped CdS/ZnS core/shell QDs, compared to the absence of hot electron current from undoped CdS/ZnS core/shell QDs. The hot electron photoemission current increases quadratically to the excitation intensity consistent with biphotonic Auger upconversion mechanism. In Figure 3c, the dependence of the hot electron photoemission current on the electrical bias between the photocathode and anode is shown. The photoemission current detected at negative biases, which repel the ejected hot electrons from reaching the anode, depends on the kinetic energy of the ejected hot electrons. The stopping voltage (approximately −0.4 V in Figure 3c) can be interpreted as the upper limit of the kinetic energy of hot electrons generated from Mn-doped CdS/ZnS core/shell QDs that can be measured by using the setup shown in Figure 3a. Ideally, kinetic energy spectrum of the entire hot electron population is needed for more complete understanding of the energetics and dynamics of hot electron transfer. Presently, there is no study reporting the full excess energy spectrum of the upconverted hot electrons, and only the average kinetic energy can be estimated. Time- and energy-resolved photoelectron emission measurements under pulsed excitation conditions may provide some of the necessary information.

**Figure 3:**
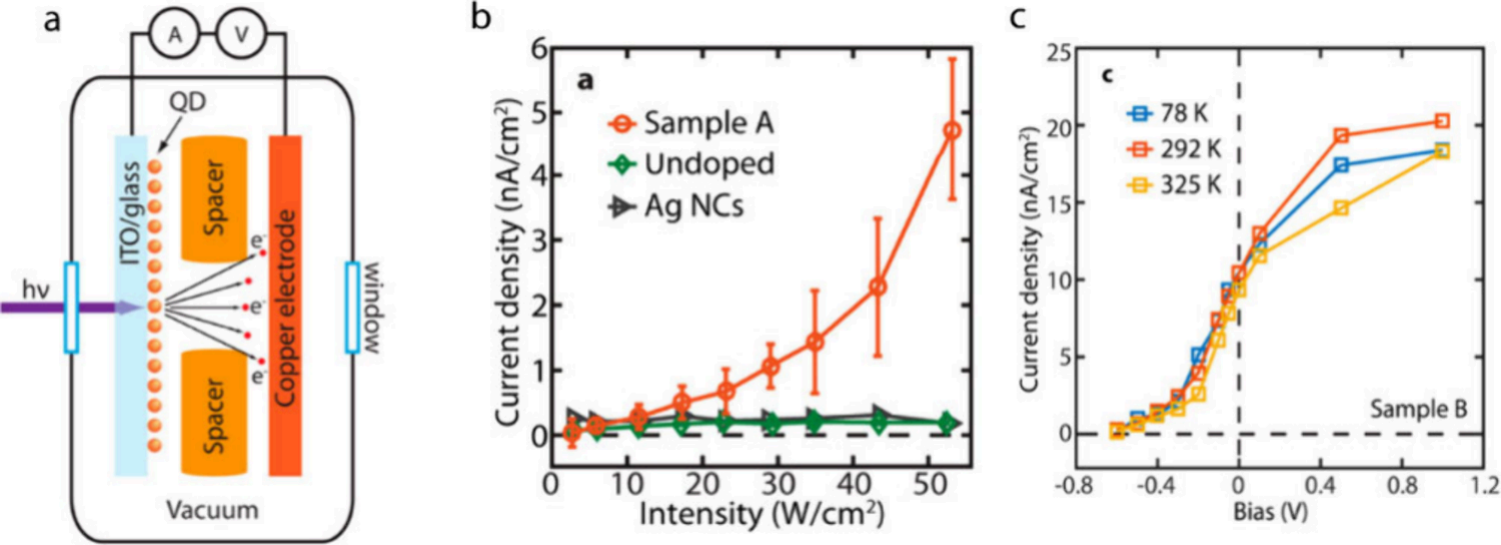
(a) Diagram of the setup measuring the photoemission current of the Auger-upconverted hot electrons in vacuum. (b) Hot electron photoemission current density measured from Mn-doped CdS/ZnS (orange), undoped CdS/ZnS (green), and silver (gray) nanocrystals. (c) Bias-dependent photoemission current density of Mn-doped nanocrystals. Adapted from ref 47 (Copyright 2016, American Chemical Society).

Figure 4 compares hot electron photoemission current from Mn-doped CsPbBr_3_ QDs and nanoplatelets (NPLs) as a function of excitation intensity, and bias applied between the two electrodes.^59^ Because of the significantly stronger quantum confinement in NPLs (∼2 nm thick) than in QDs (∼6 nm), exciton-Mn energy transfer is much faster in NPLs than in QDs for a comparable doping concentration. This difference is responsible for the faster saturation of the population of the excited Mn^2+^ with increasing excitation intensity, resulting in the earlier onset of a linear increase of photocurrent vs excitation intensity in NPLs. Another difference between the QDs and NPLs is that the average energy of the photoemitted hot electrons is ∼ 0.15 eV higher in NPLs, reflected in the shift of the bias-dependent photocurrent (Figure 4c and d). This energy difference corresponds to the higher quantum-confined electron level in the NPL than in the QDs, showing the possible structural control of the upconverted hot electrons’ kinetic energy spectrum.

**Figure 4:**
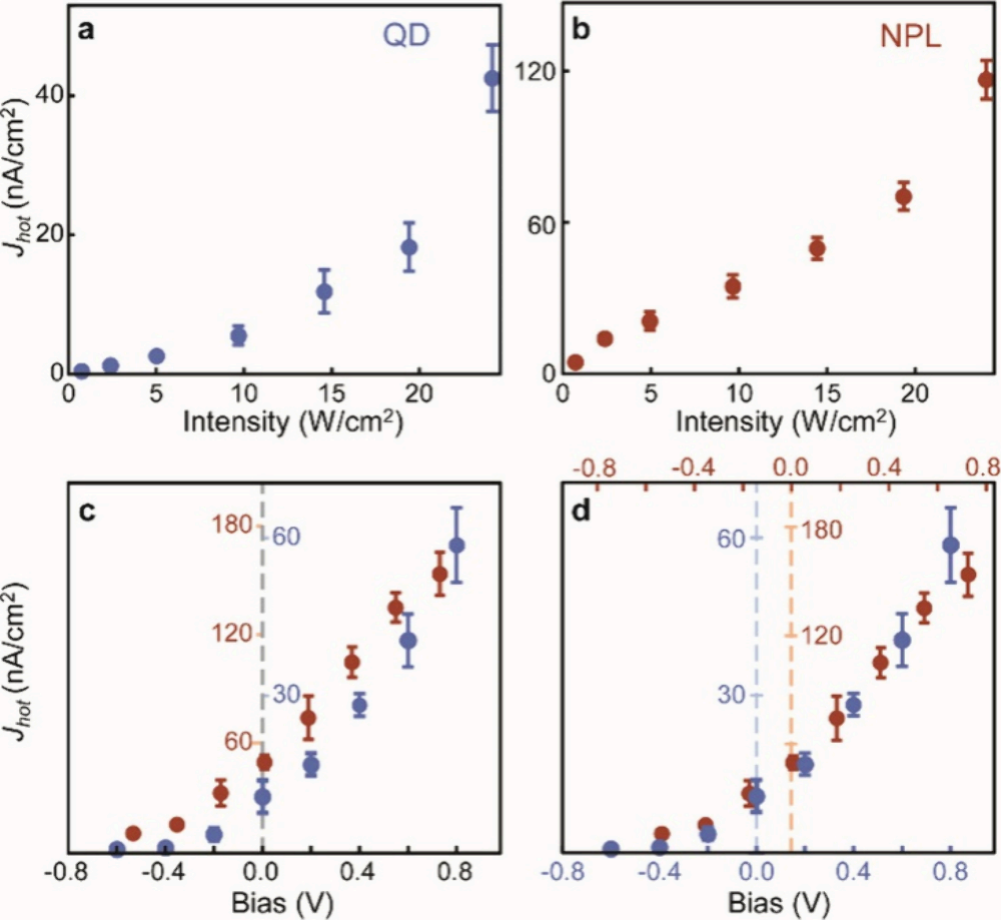
(a, b) Intensity-dependent hot electron photoemission of Mn-doped CsPbBr_3_ QDs (a), and Mn-doped CsPbBr_3_ NPLs (b). (c) Comparison of the bias-dependent photoemission current densities of Mn-doped QDs (blue) and NPLs (red). (d) Comparison of bias-dependent photoemission current density of Mn-doped QDs (blue) and NPLs (red) with a 0.15 V relative shift of bias on the horizontal axis. Adapted from ref 59 (Copyright 2022, American Chemical Society).

In the colloidal dispersion of Mn-doped nanocrystals in liquid media, hot electrons injected into the solvent can produce solvated electrons if they possess sufficient energy to be injected into the continuum level of solvent. Since the optical absorption spectra of the equilibrated solvated electrons in various solvents are known, they can be readily detected from pump–probe transient absorption measurements. However, short solvated-electron lifetimes generally preclude optical detection under cw excitation for many solvents. The optical detection of the solvated electron formed from hot electrons generated in QDs was first made in aqueous dispersion of undoped CdS QDs and subsequently in CdSe QDs under direct resonant two-photon excitation condition (Figure 5a,b).^77–79^ Hydrated electrons generated from the upconverted hot electrons in Mn-doped QDs were also optically detected recently via pump–probe measurements (Figure 5c).^50,61^ These time-resolved studies show that upconverted hot electrons can evolve into solvated electrons capable of driving reduction reactions in the bulk solution, in addition to performing interfacial hot electron transfer and presolvated electron-driven reduction prior to full solvation.

**Figure 5:**
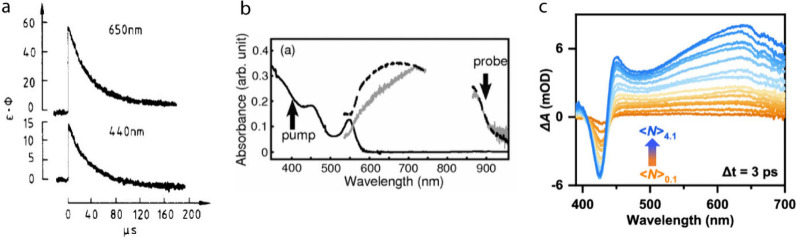
(a) Transient absorption decay signal of a hydrated electron from undoped CdS QDs. Adapted with permission from ref 78 (Copyright 1988, American Chemical Society). (b) Absorption spectrum of hydrated electron (solid gray line) from undoped CdSe QDs (solid black line), acquired 1 ns after excitation. Adapted with permission from ref 79 (Copyright 2004, American Physical Society). (c) Pump-intensity dependent TA spectra of Mn-Doped nanoparticles acquired 3 ps after 330 nm excitation. The broad positive feature at ∼650 nm corresponds to the absorption spectra of solvated electrons. Adapted with permission from ref 61 (Copyright 2025, American Chemical Society).

For photochemical applications of the Auger upconverted hot electrons, the key metric is the efficiency of generating “usable” hot electrons. However, their rapid evolution through multiple competing pathways makes this quantification challenging. These pathways include cooling, interfacial transfer, injection and solvation in the solvent, geminate recombination, and diffusion-controlled reactions with acceptors (Figure 6)

**Figure 6:**
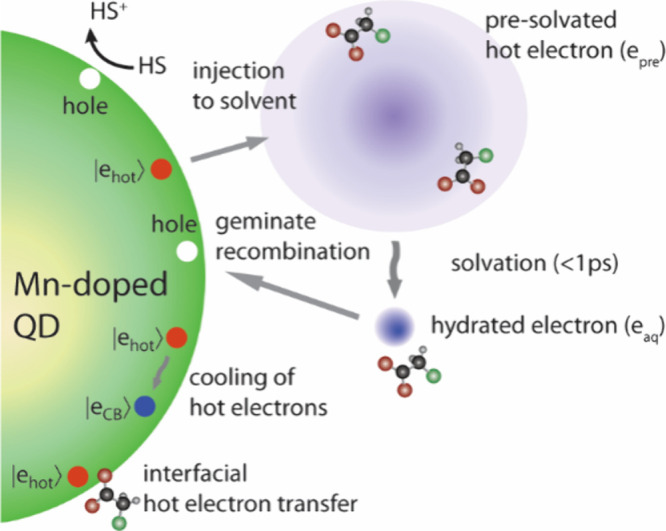
Diagram of possible reaction pathways of photogenerated hot electrons. Adapted with permission from ref 21 (Copyright 2025, American Chemical Society).

So far, two different approaches have been used to address the problem of determining the efficiency of producing hot electrons, especially in colloidal dispersion in a liquid solvent. One is optical detection of solvated electrons formed from the hot electrons in the absence of added electron acceptors via pump–probe transient absorption. Although this method only detects fully equilibrated solvated electrons and is limited to pulsed excitation, it still provides a systematic way to compare photon-to-solvated electron quantum yields across materials.^80–83^ Several groups have reported the quantum yield of producing hydrated electrons from the upconverted hot electrons in Mn-doped nanocrystals under pulsed multiexciton excitation condition. Livache et al. determined the internal quantum efficiency (IQE, number of solvated electrons detected/number of photons absorbed) of producing hydrated electrons from Mn-doped CdSe/CdS core/shell QDs employing pump–probe transient absorption. They reported IQE of ∼3.5% under visible light excitation (2.4 eV) and ∼11% under ultraviolet (UV) excitation (3.6 eV) at the excitation density of ∼30 per QD for both visible light and UV excitations.^50^ Yao et al., who performed similar experiment on Mn-doped ZnSe QDs, reported maximum IQE of ∼15% for hydrated electron generation under UV excitation (330 nm, 3.8 eV) at the excitation density of 20 per QD.^61^ Because Mn-mediated hot electron upconversion is a nonlinear sequential multiphoton process, solvated electron yields increase superlinearly with the excitation density under pulsed conditions. As a result, quantum yields measured under multiexciton pulsed excitation must be interpreted cautiously when compared to cw excitation with very low exciton densities.

The second method of quantifying usable hot electrons is using the hot electron- and solvated electron-selective “indicator” reaction. Unlike in optical detection of solvated electron, hot electron- and solvated electron-selective reaction captures not only the solvated electrons but also hot electrons inside Mn-doped nanocrystals and presolvated electrons in solvent, while excluding lower-energy bandedge electrons. Orrison et al. used the reduction of monochloroacetate (MCA) as the ‘indicator’ reaction to determine the quantum efficiency of generating hot electrons and solvated electrons from Mn-doped CdSSe/ZnS core/shell QDs in aqueous media in the presence of sacrificial hole scavenger.^21^ MCA reduction irreversibly cleaves a C–Cl bond to release Cl^–^, and its reduction potential (−2.7 V vs NHE) lies just below that of the hydrated electron (−2.9 V vs NHE), making it a selective probe of highly reducing electrons. Because the initially produced hot electrons and presolvated electrons are more reducing than solvated electrons, MCA reduction reports on a broad ‘usable’ subset of the hot electron population. The pathways of MCA reduction via interfacial hot electron transfer to surface-bound MCA, reduction by presolvated electron via static quenching, and reduction via bimolecular collision between the solvated electron and MCA are illustrated in Figure 6.

Under cw visible light (455 nm) excitation at ∼0.1 mW/cm^2^, the quantum yield of producing Cl^–^ as the product of MCA reduction (QY_prod_) via the biphotonic process was reported as high as ∼40% (in terms of IQE, ∼20%), which is remarkably high considering the relatively low intensity of cw visible excitation. Since the value of QY_prod_ saturated at the higher concentration of MCA, its saturation value was taken as the lower limit of the quantum efficiency of generating hot electrons and solvated electrons at a given excitation intensity and solvent environment. Since hot electron generation under cw excitation requires continuous removal of photogenerated holes, both the efficiency and mass transport of the sacrificial scavenger at the QD interface can become rate-limiting at high excitation intensities. Because MCA is an effective reporter of the amount of hot electrons generated under a given excitation condition in aqueous media, comparing its reaction yield with that of the target reactant can enable the estimation of what fraction of the initially generated hot electrons is used for reducing the chosen reactant.

An interesting observation in quantification of QY_prod_ using MCA reduction is its dependence on photoexcitation history, in which QY_prod_ diminishes over ∼30 min to a lower steady-state value although it recovers when excitation is interrupted and resumed. It was hypothesized that slow photoinduced accumulation of trapped negative charges on Mn-doped QDs reduces QY_prod_ by decreasing the local concentration of negatively charged MCA near the QD surfaces. These observations show that the usable hot electron yield is dictated by a delicate interplay among ultrafast Auger processes, interfacial charge transfer, solvation dynamics, and long-time scale charge accumulation, indicating the need for better mechanistic understanding across these coupled time and length scales.

Upconverted hot electrons in Mn-doped semiconductor nanocrystals been utilized for various photocatalytic reactions due to their high thermodynamic driving force, long-range transfer capabilities, and ability to produce solvated electrons. The first group of reactions that demonstrated the benefits of the upconverted hot electrons includes hydrogen evolution reaction (HER) and carbon dioxide (CO_2_) reduction. In a study by Dong et al., HER in water was performed using Mn-doped CdSSe/ZnS core/shell QDs under cw excitation with a xenon lamp.^19^ Mn-doped CdSSe/ZnS QDs exhibit higher H_2_ evolution rates than undoped QDs under cw excitation, and the ratio of H_2_ evolution rates between Mn-doped and undoped QDs increases linearly with intensity before saturating, consistent with a biphotonic Mn-mediated Auger mechanism (Figure 7a,b). Although Mn-doped QDs generate fewer total electrons than undoped QDs, their higher H_2_ evolution rates arise from both the larger reduction potential and the long-range transfer capacity of the hot electrons.

**Figure 7:**
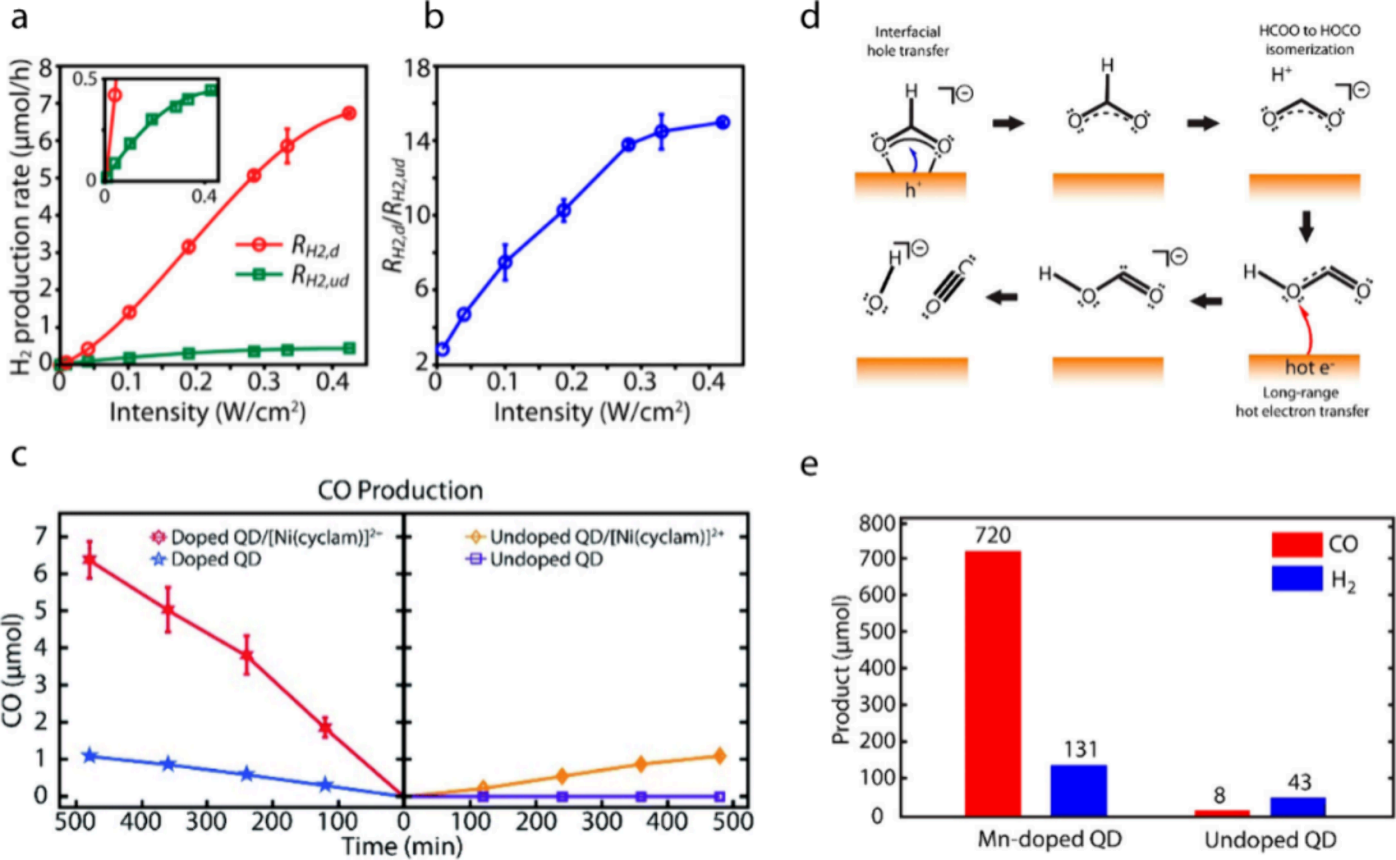
(a) H_2_ production rates as a function of light intensity of Mn-doped (red) and undoped (green) nanocrystals. (b) Ratio of H_2_ production rates by Mn-doped (R_H2,d_) and undoped (R_H2,ud_) nanocrystals as a function of light intensity. Adapted with permission from ref 19 (Copyright 2015, American Chemical Society). (c) CO production as a function of reaction time for four different combinations of hybrid photocatalysts. Adapted with permission from ref 22 (Copyright 2020, the Royal Society of Chemistry). (d) Proposed mechanism for photocatalytic conversion of formate to CO via sequential hole transfer and hot electron transfer by Mn-doped semiconductor nanocrystals. (e) Amount of CO and H_2_ produced from formate after two h of reaction by Mn-doped (left) and undoped (right) nanocrystals. Adapted from ref 20 (Copyright 2021, American Chemical Society).

In a work by Parobek et al., the aqueous-phase photocatalytic reduction of CO_2_ to CO was performed using a hybrid photocatalyst composed of the molecular catalyst [Ni­(cyclam)]­[BF_4_]_2_ that selectively converts CO_2_ into CO and Mn-doped CdSSe/ZnS core/shell QDs that sensitize the molecular catalyst.^22^ Unlike in typical QD-molecular hybrid catalysts that require a chemical linkage between them for efficient sensitization, a simple mixture of Mn-doped CdSSe/ZnS core/shell QDs and [Ni­(cyclam)]­[BF_4_]_2_ already functions efficiently due to long-range hot electron transfer. Figure 7c compares CO production rates from four different combinations of catalysts under the same excitation conditions (0.1 W/cm^2^ at 455 nm): undoped QDs, Mn-doped QDs, mixture of undoped QDs and [Ni­(cyclam)]­[BF_4_]_2_, and mixture of Mn-doped QDs and [Ni­(cyclam)]­[BF_4_]_2_. Under identical conditions, Mn-doped QDs produce ∼ 6 times more CO than undoped QDs in the presence of [Ni­(cyclam)]^2+^ (Figure 7c), attributed to the long-range sensitization of [Ni­(cyclam)]^2+^ by hot electrons.

Orrison et al. showed that efficient conversion of formate (HCOO^–^) to carbon monoxide (CO) can be achieved using Mn-doped CdSSe/ZnS core/shell QDs, where only hot electrons could efficiently reduce the intermediate radical species formed during the reaction.^20^ The photocatalytic conversion of format into CO requires the initial hole transfer to oxidize formate into an HCOO· radical. Subsequently, the electron transfer to HOCO^•^ formed after the isomerization of HCOO^•^ is considered to produce CO and OH^–^ via decomposition of HOCO^–^ as illustrated in Figure 7d. Because of the weak surface-binding affinity expected from the HOCO^•^ radical, interfacial electron transfer cannot efficiently perform the second step of the sequential charge transfer reactions. In contrast, long-range hot electron transfer can reduce HOCO^•^ without requiring them to adsorb on the nanocrystal surface, resulting in 2 orders of magnitude enhancement in the efficiency of conversion to CO as shown in Figure 7e. The internal quantum yield of CO production by Mn-doped QDs was ∼10% to this two-step redox reaction, which is 2 orders of magnitude higher than that with undoped QDs. These findings indicate that upconverted hot electrons can participate beneficially at several key stages of a reaction, such as catalyst sensitization, initial reactant reduction, and intermediate reduction, while sustaining practically useful quantum yields of reaction.

Recently, the photocatalytic application of upconverted hot electrons was expanded beyond simple reduction reactions, demonstrating their versatility in wide range of organic transformations as summarized in Figure 8.^23^ In a study by Cao et al., Mn-doped CdS/ZnS QDs were shown to efficiently dehalogenate aryl halides with very negative reduction potentials (−3.4 V vs Standard Calomel Electrode (SCE)) under low-intensity blue light excitation in a polar organic solvent (e.g., 5 mW cm^–2^). The same study further demonstrated the upconverted hot electrons’ activity in other more complex organic reactions including Birch-type reductions, reductive σ-bond cleavages, and a range of cross-coupling reactions (C–C, C–S, C–B, C–Sn). Mechanistic analysis proposes that the key single-electron transfer (SET) step can be carried out by either an upconverted hot electron or a solvated electron generated from it, indicating that both interfacial and solution-phase electron transfer pathways may operate simultaneously providing mechanistic flexibility.

**Figure 8:**
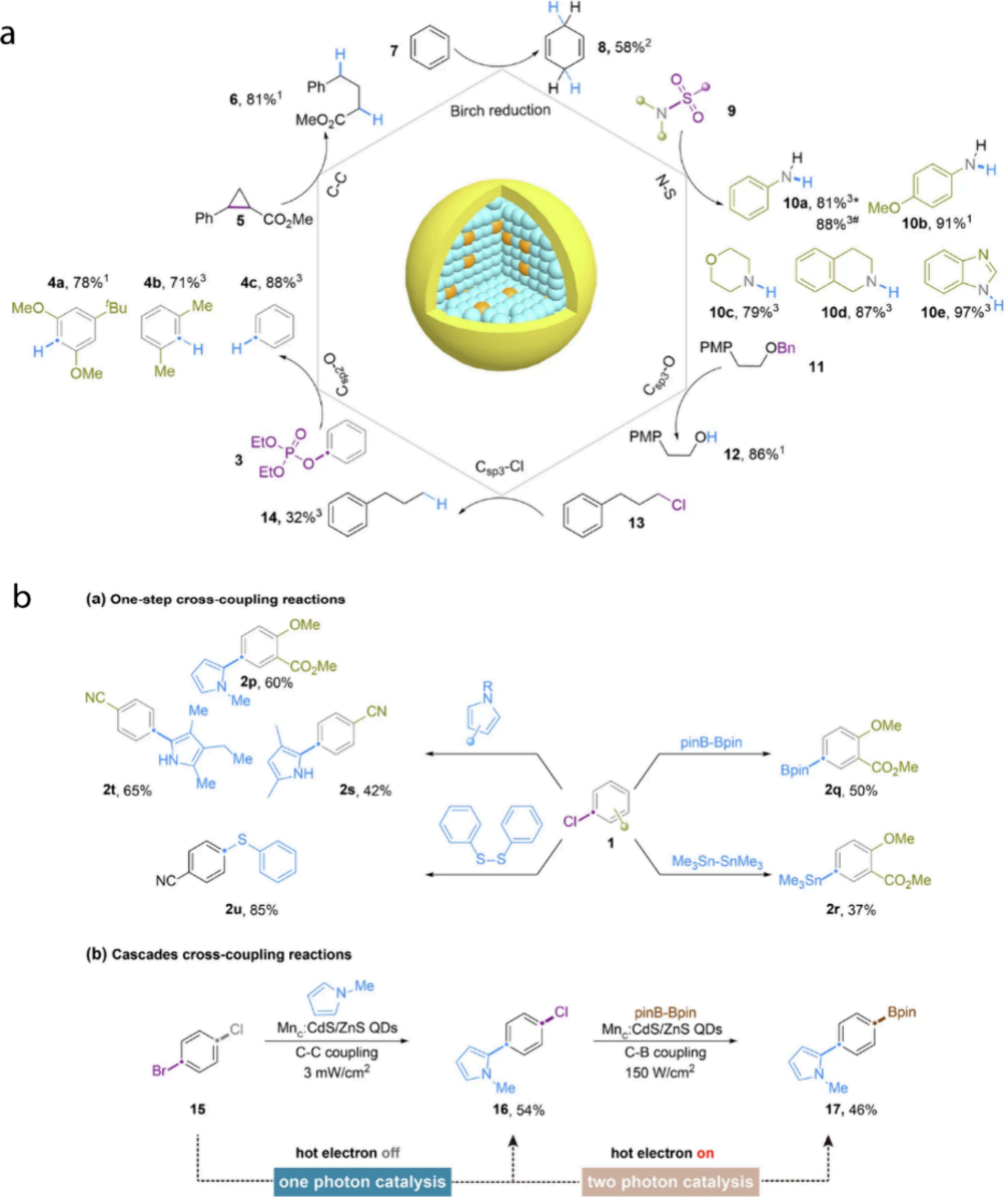
(a) Scheme showing various reductions performed by Mn-doped CdS/ZnS QDs. (b) Scheme of various one-step and multistep cross-coupling reactions performed by Mn-doped CdS/ZnS QDs. Adapted with permission from ref 23 (Copyright 2025, Springer Nature).

All these studies demonstrate that Auger-upconverted hot electrons can efficiently drive a wide variety of photocatalytic transformations, ranging from proton reduction and CO_2_ conversion to more complex multielectron organic reductions. The results clearly reveal several key advantages of the upconverted hot electrons, including their exceptionally strong reducing power, their ability to transfer over long distances beyond the nanocrystal surface, and their capability to generate solvated electrons that can enable the reaction within the solution. Despite these promising demonstrations, quantitative information on the quantum yields of hot electron-enabled reactions, both for Mn-mediated upconversion systems and for hot electrons produced through other mechanisms, remain scarce. Such data will be essential for establishing meaningful benchmarks, comparing different hot electron generation strategies, and guiding the rational design and optimization of the hot electron-based photocatalysts.

Despite these advances, several crucial knowledge gaps remain. Quantitative understanding of hot electron generation efficiency correlated with the host material, excess kinetic energy distribution of hot electrons, and development of robust, standardized protocols for determining the quantum yields of hot electron-specific reactions are still lacking. Even in systems where an internal quantum yield has been reported, the relative contributions of different reactive electron species remain unclear. In aqueous media, earlier studies have shown that quasi-free electrons, although surviving for only several hundred fs, are capable of inducing long-range reduction on ultrafast time scales, followed by a slower reduction phase governed by hydrated electrons and diffusion-limited transport.^84^ This indicates that the relative roles of different reactive species can vary significantly depending on the solvent medium that determines the lifetimes of both quasi-free and solvated electrons. Future studies that can distinguish these different reaction pathways will be essential for drawing a complete mechanistic picture of the upconverted hot electron-driven chemistry. For instance, controlling the ligand on the QD surface that can control the surface binding affinity of the reactant may shed more light on disentangling the contribution of direct interfacial hot electron transfer vs presolvated and solvated electrons. The contribution of presolvated electrons, which will be substantial only at relatively high concentrations of the reactant, can be deduced from examining the static scavenging of the reactant by the excited state of solvated electron that mimics delocalized presolvated electrons. In parallel, advances in materials synthesis that improve the efficiency of hot electron generation, along with better control of the interfacial and solvent environments that optimize the reaction kinetics, will be important to broaden the impact of the upconverted hot electrons for visible-light driven chemical transformations.^85,86^
